# The Clinical Management of Traumatic Palatal Ulcers in an Adolescent Patient: A Common Lesion in Mini-Implant-Assisted Rapid Maxillary Expansion

**DOI:** 10.3390/medicina60111784

**Published:** 2024-10-31

**Authors:** Cristopher Barriga, Gonzalo Muñoz, Paulo Sandoval, Alejandro Lara, Flavio Copello

**Affiliations:** 1 Residency Program in Orthodontics and Dentofacial Orthopedics, Universidad de La Frontera, Temuco 4780000, Chile; cabarrigas@gmail.com; 2 Undergraduate Research Group in Dentistry (GIPO), School of Health Sciences, Universidad Autónoma de Chile, Temuco 4780000, Chile; 3 Department of Pediatric Dentistry and Orthodontics, School of Dentistry, Universidad de La Frontera, Temuco 4780000, Chile; paulo.sandoval@ufrontera.cl (P.S.); alejandro.lara@ufrontera.cl (A.L.); 4 School of Dentistry, University of Maryland, Baltimore, MD 21201, USA; fcopello@umaryland.edu

**Keywords:** rapid maxillary expansion, MARPE, traumatic palatal ulcers

## Abstract

*Background*: This case report describes the clinical management of a traumatic palatal ulcer, a complication associated with mini-implant-assisted rapid maxillary expansion (MARPE). *Case Report*: A 13-year-old female patient with maxillary constriction underwent MARPE treatment using a custom acrylic expander anchored by four mini-implants. Despite proper planning and device design, the patient missed her first follow-up appointment and continued activations, resulting in tissue inflammation and embedding of the device. Upon examination, swelling and displacement of the cement were observed, necessitating device removal. The traumatic ulcer was treated with chlorhexidine gel, paracetamol, and a soft diet. Complete recovery was achieved within one month. A second expander was then designed and installed, with more frequent monitoring and improved hygiene protocols. This approach led to successful expansion without complications. This case highlights the importance of precise treatment planning, proper mini-implant selection, and regular follow-ups in MARPE therapy. It also emphasizes the need for patient compliance and effective hygiene measures to prevent complications. *Conclusions*: The successful management of the traumatic ulcer demonstrates that prompt action and consideration of cost-effective treatment options can lead to positive outcomes in addressing MARPE-related complications.

## 1. Introduction

Maxillary constriction is a developmental condition that must be addressed promptly upon diagnosis. If left untreated, it can lead to more challenging clinical conditions as the subject ages. Rapid maxillary expansion (RME) is a viable and efficient alternative to correct mild-to-moderate maxillary constriction during growth by taking advantage of the bone plasticity of the different sutures of the craniofacial complex [1]. Given that the maxilla is composed of two bone segments joined by a suture, it can expand depending on the plasticity of these structures.

The midpalate suture has different degrees of ossification depending on the stage of bone maturation. These stages of ossification are classified according to the morphology of the midpalate suture on a cone-beam computed tomography (CBCT) image. It can also be associated with the stage of bone maturation according to the development of the first three cervical vertebrae [2,3,4].

An alternative for treating maxillary constriction in individuals with a higher degree of ossification of the sutures is mini-implant-assisted rapid palatal expansion (MARPE). This technique involves using an expander device anchored to both sides of the midpalate suture by four mini-implants. The current literature shows that the MARPE technique has proven safe and effective for treating moderate and severe maxillary atresia [1,5].

Additionally, this technique has been associated with several benefits, such as increased upper airway volume and substantial improvements in breathing in patients with obstructive sleep apnea, significantly improving their quality-of-life indices [5].

The first to report this technique was Kee Joon Lee in 2010, who introduced the use of a modified Hyrax-type appliance with welded rings to support and guide the installation of four mini-implants (MI), considering two MI mesially and two distally to the expander device [6,7]. Since then, many other devices have been designed, expanding the alternatives for this type of treatment.

The acrylic mini-implant-supported expander is a more economical alternative to the conventional MARPE devices. It consists of a central expander screw with an acrylic body with four perforations for the MI. One study described better expansion results than other types of MARPE devices [8].

Some considerations should be observed when planning the palate expander in general. Specific protocols must be followed to ensure the stability of the MI, which must be selected individually based on bone thickness, soft tissue thickness, and the distance between the expander and the palate bone [9]. On the other hand, a fundamental parameter to ensure the stability of the appliance and achieve better expansion results is the bicortical anchorage, which must be measured and evaluated individually, ensuring that the length of the selected MI will reach both the cortical bone areas of the palate bone [10,11].

Despite the operator’s technical expertise, this technique is not error-free, and the literature has documented some issues and complications related to the MARPE technique [12,13], among which we can mention bone defects between central incisors, anterior gingival recessions, irreversible pulpitis, dental lateral tipping, thinning of the labial/buccal bone, significant mobility of the anterior teeth, soft tissue inflammation, hypertrophy/hyperplasia of the palatal mucosa associated with ulcerations, erythema, itching, and discomfort in the area [14,15].

The aim of this case report was to describe and discuss the undesirable effects during the performance of the MARPE technique: traumatic palatal ulcer and its clinical management.

## 2. Case Report

The subject of this case report was recruited from the Orthodontics and Dentofacial Orthopedics clinic at the Universidad de La Frontera, Chile. Prior to this publication, the guardian signed the informed consent form, and subsequently, the subject signed an informed consent document.

The subject was a 13-year-old female who came for a clinical evaluation referred by her general dentist. The guardian said that the subject had previously received interceptive orthodontic treatment with removable appliances. During the clinical examination and medical history, it was reported that the patient was under treatment for allergic rhinitis.

**Figure 1:**
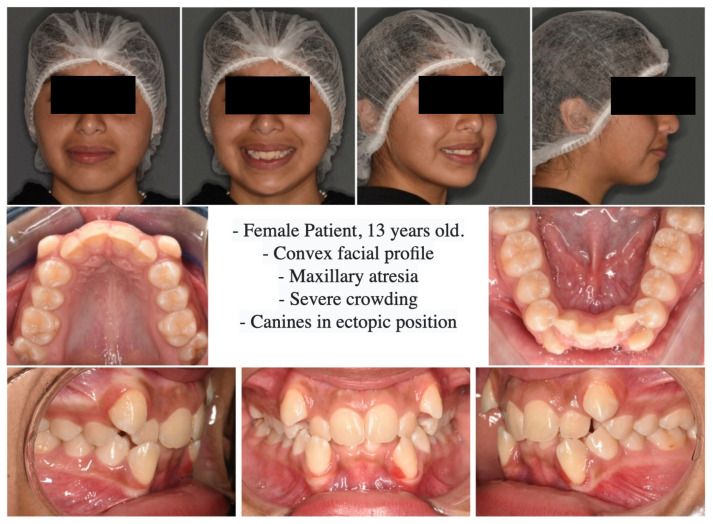
Initial diagnostic photographs for orthodontic treatment.

**Figure 2:**
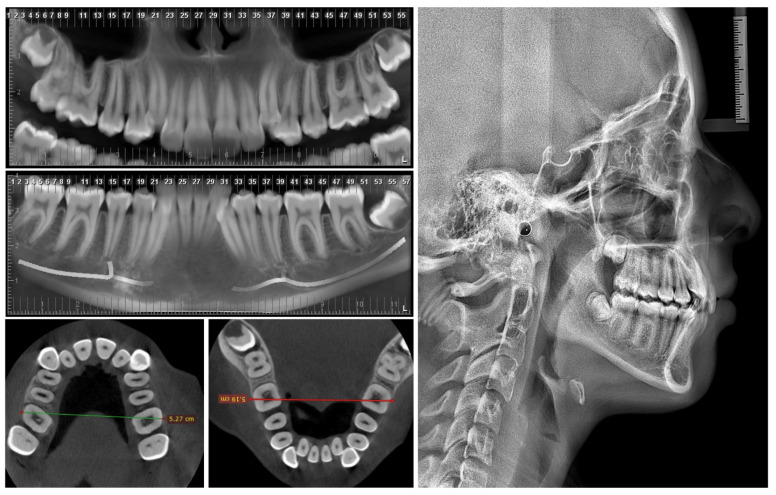
Diagnostic radiographs and initial CBCT scans.

On extraoral analysis, a dolicofacial biotype was observed, with a moderately enlarged lower third and a convex profile. The intraoral analysis showed incomplete permanent dentition, Angle molars Class II on the right and Class I on the left. In addition, severe upper and lower crowding, upper and lower canines in an ectopic position, and maxillary constriction were observed. (Figure 1 and Figure 2).

The therapeutic approach in this case was non-conservative and divided into two stages. First, there was a phase of maxillary expansion assisted with mini-implants to resolve the horizontal discrepancies, and subsequently, there was a second phase with the extraction of four premolars to resolve dental crowding.

**Figure 3:**
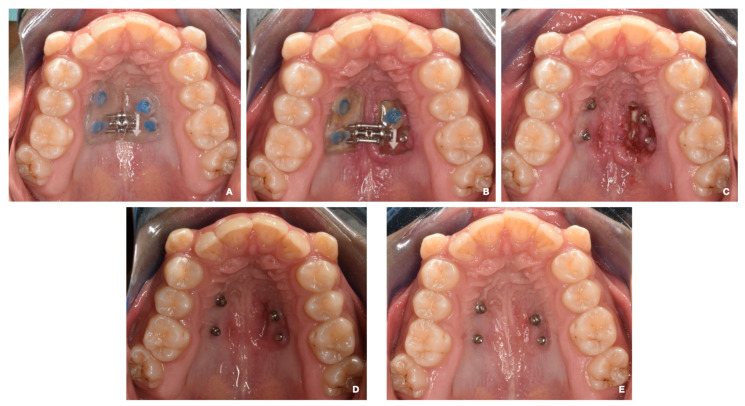
(**A**) The installation of a MARPE apparatus. (**B**) Day 14. First clinical check-up. The expander is embedded in the palatal mucosa on the left side. (**C**) The device is removed, and an ulcer with purulent exudate is observed in the area under the expander. Pharmacotherapy started. (**D**) A check-up at 7 days with pharmacotherapy. The use of 0.12% chlorhexidine is maintained for two more weeks. (**E**) A check-up at 30 days. The mucosa is in good condition with total remission of the lesion.

Subsequently, a maxillary impression was performed to fabricate the acrylic device, which was cemented to the MIs using Ultra Band-Lok resin (Relience Orthodontic) (Figure 3A).

The activation protocol was started: 2 activations of ¼ mm per day for 7 days performed by the guardian, with weekly follow-up.

The patient missed the first follow-up, and it was not possible to reach the guardian until the 14th day after the MARPE installation. On that day, the guardian called the office stating that his daughter had pain in the palatal area. It was recommended that the activations be stopped, and that the patient should be seen as soon as possible.

On the 20th day of treatment, the patient was seen, and swelling was observed on the left side of the MARPE appliance, as well as the displacement of the cement in relation to the MI on the same side. In addition, the expander was embedded by soft tissue in that area. The device was removed and cleaned by irrigation with saline solution and 0.12% chlorhexidine. 

An ulcerative and erythematous lesion of approximately 12 mm in diameter, which showed the presence of purulent exudate and is associated with acute pain, was observed. The patient was prescribed 1% chlorhexidine gel (topical application on the affected area 3 times a day for 7 days), 1 g paracetamol (1 tablet every 12 h for 5 days), and a soft diet (preferably cold for 5 days). A conversation was held with the guardian about what happened, and a follow-up appointment was scheduled in 7 days (Figure 3B,C).

The lesion was in remission at the follow-up appointment, with the neoformation of tissue in the area and no pain. A follow-up appointment was performed 1 month after the lesion started, and the complete recovery of the area was noted. During this appointment, a new impression was obtained for the second palate expander (Figure 3D,E).

**Figure 4:**
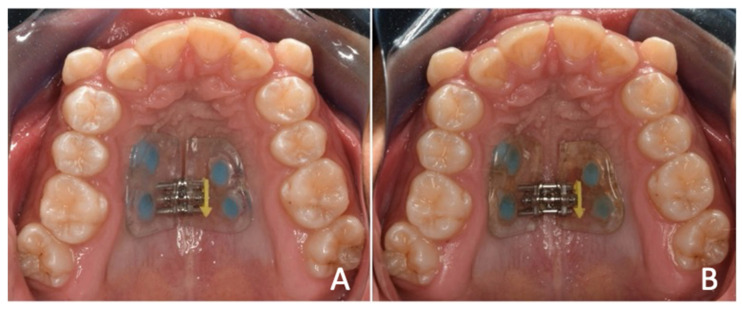
Second expander. (**A**) Cementing of expander. (**B**) Check-up at 14 days.

A second expander was designed on the same initial 4 MS. The same initial design parameters were followed, and it was cemented approximately one month after total healing of the traumatic ulcer. Two activations per day were prescribed (one in the morning and one in the evening) every day for 14 days. Monitoring and hygiene were performed twice a week during the active phase of the treatment (Figure 4).

On day 14, expansion was observed within the desired parameters to correct the transverse discrepancy, and there was a diastema of approximately 1 mm between the central incisors. The screw was stabilized with a composite, and the patient went to the retention phase, which would take from 4 to 6 months.

## 3. Discussion

There are currently different techniques to perform the transverse correction of the median palatal suture. The results obtained by Angelieri et al. (2013) demonstrated that it is possible to establish the degree of maturation of the maxilla [2]. Thus, this information can be used to determine which technique can be applied with the greatest precision, obtaining a predictable and efficient result in the opening of the median palatal suture, and determining the best device and/or technique for use in each case. These devices can range from conventional expanders (such as Hyrax, McNamara, and Hass) to MS-anchored expanders (such as the Maxillary Skeletal Expander (MSE) device or custom-made acrylic devices) and, finally, surgically assisted expansion. However, there is currently no clear relationship between the maturation stage of the median palatal suture and successful expansion. Oliveira et al. (2021) showed a negative correlation between age and maxillary expansion, reporting that success rates decrease considerably with increasing age. On the other hand, all the cases in which miniscrew-assisted expansion failed in their study were in the maturation stages D and E; therefore, expansion should be performed surgically in those cases [16]. From this point of view, this classification becomes an important starting point when defining the type of treatment to be performed. According to radiographic analysis, the patient in this study was in Angelieri stage C, so the patient was within the group of individuals for whom the miniscrew-assisted technique is indicated [2,3].

Although maxillary expansion is a stable technique in the long term [17,18], recurrence of a percentage of expansion is a reality when rapid maxillary expansions are performed with or without miniscrews. Chun et al. (2022) reported similar results in conventional and mini-implant-assisted expansion; however, both the recurrence and impact on other tissues were shown to be considerably lower in those individuals in whom MARPE was performed, reinforcing the fact that even though expansion may be successful conventionally or with mini-implants, MARPE results appear to be more stable and predictable in the long term and have less impact on the supporting tissues [19,20].

Different clinical and/or radiographic methods can determine transverse discrepancies. The Ricketts PA cephalometric analysis uses frontal teleradiography and is based on the dimensions of the maxillae compared to a table of age-adjusted normative values [21]. The evaluation using Andrews’ element III is based on the transverse correlation of the maxilla and mandible by taking bone and dental reference points (WALA—WALA in the mandible, FA—FA in the maxilla) from the measurement of dental study models [22,23]. Tamburrino et al. (2010) proposed a CBCT-based analysis, as this can visualize bone margins and dental structures without the distortion of frontal teleradiography and can also make it possible to assess hard tissues and dental inclinations not visible in the analysis of the models [24]. In this case, we used the method of Tamburrino et al. (2010), since the same CBCT test would be used to plan the miniscrews. On the other hand, it is important to note the need for more studies correlating the different methods to validate this technique clinically.

It is important to note that the selection of the mini-implants is a fundamental point in the application of this technique. The stability of the mini-implants must be sufficient for the active treatment and ensure its duration until the stabilization phase. Naveda et al. (2022) showed that the mineralization values of the median palatal suture after treatment take a considerable time to return to normal, so maintaining the anchorage devices is essential to avoid recurrences during this period [25]. Nojima et al. (2018) provided a clear protocol for proper miniscrew selection in the MARPE technique, which includes measuring the soft tissue width, the distance of the device to the back of the palate, and the thickness of the palatine bone considering 1 mm bicorticality [9], as this is essential to avoid damage to the trabecular bone and to maintain the long-term stability of the mini-implants, as indicated by Copello et al. (2021) and Li et al. (2022) [10,12].

Mohan et al. (2022) showed the importance of the length and thickness of the mini-implants to the stability, obtaining a significantly lower displacement with the longer and thicker miniscrews [26]. Following the previous recommendations, in this clinical case, 1.8 mm diameter miniscrews were selected, the angulation was increased to obtain a larger insertion surface and to enable the installation of longer mini-implants, self-drilling mini-implants were used, and their planning and selection were carried out to obtain 1 mm of bicorticality, which was also radiographically controlled using CBCT. In the miniscrews installed at the molar level, the contact of the miniscrews with the medial cortex of the maxillary sinus was also observed (Figure 3); hence, this anchorage may be considered tricortical. Although logical thinking would indicate that this would increase stability, studies should be conducted to evaluate the stability of tricortical miniscrews compared to mono- and bicortical ones.

The appliance was designed with a straightforward insertion and removal axis. It contained an adequate amount of acrylic to withstand the force exerted by the expander screw on the mini-implants. Additionally, the appliance was securely attached to the screws using adhesive cementation. The acrylic of the external area of the mini-implant was approximately 2 mm to facilitate the removal of the appliance. The area in contact with the palate was situated entirely on the hard palate and was completely smooth. There was no noticeable ischemia during the cementation process. The edges were carefully rounded and polished to prevent it from becoming embedded. The expansion screw was placed between the anterior and posterior miniscrews. Despite the limited evidence available on the use of such devices, Gupta et al. (2023) pointed out in their finite element study that these devices showed better expansion results than conventional devices [8].

Pérez-Flores et al. (2020) stated that during orthopedic treatment with maxillary expansion appliances, patients cannot achieve proper hygiene and effective plaque control, resulting in moderate to severe gingival inflammation [27]. According to Rosa et al. (2005), it is possible that after removing orthopedic devices with an acrylic palatal cover, a transient bacteremia may occur; therefore, a careful approach is recommended when treating patients with this type of device [28]. Following the above recommendations, treatment check-ups were set for every 7 days during the active phase of treatment to monitor hygiene in the appliance area and soft tissue health, and every 1 month in the stabilization period to clean the appliance area. However, the patient did not attend her first check-up but continued with the activations, which we believe was the factor that triggered the tissue inflammation and subsequent embedding of the device.

In this regard, hygiene is fundamental to avoiding complications when using this type of device. As observed clinically, this was the fundamental difference between the first device that failed and the second one that succeeded in expanding without complications. To maintain the hygiene and health of the supporting tissues, we irrigated with 0.12% chlorhexidine during the activation process at least once a week on the surface under the acrylic and prescribed the use of a daily oral irrigator in the patient’s normal hygiene routine for the mechanical removal of food. In the stabilization phase, we irrigated the area under the appliance once a month, maintaining the use of the oral irrigator in the patient’s daily hygiene.

Colak et al. (2021) and Rutili et al. (2022) have observed that both fast and slow maxillary expansion yield similar outcomes; however, slow expansion protocols are associated with less pain, tissue pressure, and adverse effects on the tooth roots [29,30]. For this reason, a protocol of ¼ mm activation of the device twice a day was selected so that strong pressure was not exerted on the tissue. However, the literature lacks evidence or information about the activation protocols in the MARPE technique related to outcomes or adverse effects.

As indicated by Kapetanović et al. (2022), moderate pain in the first week is a normal effect of the MARPE technique and tends to be well tolerated by patients, decreasing from the second week onwards [31]. From this point of view, moderate pain is not a reliable indicator of treatment failure in the first week, but it should raise concern if it persists and intensifies over time.

Regarding the treatment of this type of oral lesion, there is no specific protocol. However, using the clinical guideline proposed by Schemel-Suarez et al. (2015), we can determine the most accurate diagnosis and treatment plan for this lesion, which falls under traumatic ulcers. They advised that treating traumatic ulcers basically consists of eliminating the irritant [32]. In addition, we decided to include 1% chlorhexidine topical gel twice a day for 7 days and paracetamol 1 g every 12 h for 5 days or in case of pain. A soft and cold diet for 5 days was also recommended.

It is important to point out that the set of measures adopted to resolve the problem was simple and low-cost. They were also explained promptly to the guardian, which resulted in the patient’s good acceptance and determined the rapid resolution of the complication.

On the other hand, the treating doctor must know and explain to the patient that this technique needs to be closely monitored with a frequency of 1 week during the active phase, perform a thorough check of oral hygiene, and indicate the use of elements that reinforce hygiene to avoid the increase in the volume of the surrounding tissue and the embedding of the appliance, as well as other complications derived from a lack of control.

## 4. Conclusions

Miniscrew-assisted rapid maxillary expansion is an efficient technique for treating maxillary atresia. Its indication must be precise; therefore, the maxillomandibular discrepancy analysis on CBCT should be studied and validated, as it presents a good perspective by considering all the structures related to maxillary atresia and crossbite.

The design of the appliance and the selection of the mini-implants, as well as the location of the appliance, become the fundamental pillars in the success or failure of the application of this technique. In this regard, further studies are needed to assess the behavior of tricortically inserted miniscrews to ascertain their influence on MARPE.

Finally, the effectiveness in treating traumatic lesions relies on the patient’s commitment and the effective professional handling of such situations. Prompt action and the consideration of the best cost/benefit ratio for the patient are crucial to achieving positive outcomes.

## Data Availability

Data are contained within the article.
